# Role of the Adiponectin Binding Protein, T-Cadherin (cdh13), in Pulmonary Responses to Subacute Ozone

**DOI:** 10.1371/journal.pone.0065829

**Published:** 2013-06-06

**Authors:** David I. Kasahara, Alison S. Williams, Leandro A. Benedito, Barbara Ranscht, Lester Kobzik, Christopher Hug, Stephanie A. Shore

**Affiliations:** 1 Department of Environmental Health, Harvard School of Public Health (HSPH), Boston, Massachusetts, United States of America; 2 Department of Neurosciences, University of California San Diego, San Diego, California, United States of America; 3 Division of Pulmonary Medicine, Children's Hospital Boston, Harvard Medical School (HMS), Boston, Massachusetts, United States of America; Leiden University Medical Center, The Netherlands

## Abstract

Adiponectin, an adipose derived hormone with pleiotropic functions, binds to several proteins, including T-cadherin. We have previously reported that adiponectin deficient (Adipo^−/−^) mice have increased IL-17A-dependent neutrophil accumulation in their lungs after subacute exposure to ozone (0.3 ppm for 72 hrs). The purpose of this study was to determine whether this anti-inflammatory effect of adiponectin required adiponectin binding to T-cadherin. Wildtype, *Adipo^−/−^*, T-cadherin deficient (*T-cad^−/−^*), and bideficient (*Adipo^−/−^/T-cad^−/−^*) mice were exposed to subacute ozone or air. Compared to wildtype mice, ozone-induced increases in pulmonary IL-17A mRNA expression were augmented in *T-cad^−/−^* and *Adipo^−/−^* mice. Compared to *T-cad^−/−^* mice, there was no further increase in IL-17A in *Adipo^−/−^/T-cad^−/−^* mice, indicating that adiponectin binding to T-cadherin is required for suppression of ozone-induced IL-17A expression. Similar results were obtained for pulmonary mRNA expression of saa3, an acute phase protein capable of inducing IL-17A expression. Comparison of lung histological sections across genotypes also indicated that adiponectin attenuation of ozone-induced inflammatory lesions at bronchiolar branch points required T-cadherin. BAL neutrophils and G-CSF were augmented in *T-cad^−/−^* mice and further augmented in *Adipo^−/−^/T-cad^−/−^* mice. Taken together with previous observations indicating that augmentation of these moieties in ozone exposed *Adipo^−/−^* mice is partially IL-17A dependent, the results indicate that effects of T-cadherin deficiency on BAL neutrophils and G-CSF are likely secondary to changes in IL-17A, but that adiponectin also acts via T-cadherin independent pathways. Our results indicate that T-cadherin is required for the ability of adiponectin to suppress some but not all aspects of ozone-induced pulmonary inflammation.

## Introduction

Ozone (O_3_) is an environmental pollutant generated by chemical reactions of automobile emissions (NO and hydrocarbons) with sunlight. O_3_ acts as oxidizing agent on cell membranes and on proteins and lipids in the lung and airway lining fluid, leading to epithelial injury and an inflammatory response that includes induction of acute phase cytokines and chemokines, and neutrophil influx [1], [2], [3].

Adiponectin, an adipose-derived hormone that decreases in obesity [4], has important anti-inflammatory effects. For example, adiponectin treatment decreases endotoxin-induced pro-inflammatory cytokine expression and augments anti-inflammatory IL-10 expression in monocytes and macrophages [5], [6]. Exogenous administration of adiponectin also decreases allergic airways inflammation in mice [7]. In addition, we have previously reported that compared to wildtype (WT) mice, adiponectin deficient (*Adipo^−/−^*) mice exposed to subacute O_3_ (0.3 ppm for 24 to 72 h) have increased neutrophilic inflammation, and increased pulmonary expression of certain cytokines and chemokines, including IL-17A and G-CSF [8].

Several adiponectin binding proteins have been cloned including AdipoR1, AdipoR2, and T-cadherin (T-cad) [9], [10], all of which are expressed in the lungs [11], [12]. T-cad (cdh13 or H-cadherin) is a 95 kd glycoprotein which differs from other cadherin proteins by lacking both transmembrane and cytoplasmatic domains. Instead, T-cadherin is anchored, mainly on the apical surface of cells [13], via a glycosylphosphatidylinositol (GPI) linkage [14]. Importantly, T-cadherin primarily binds the hexameric and high molecular weight isoforms of adiponectin [10]. These are also the isoforms that dominate in the lung lining fluid [15]. In the heart, T-cadherin appears to mediate the beneficial effects of adiponectin. Following pressure overload, mice deficient in Tcadherin (*T-cad^−/−^* mice), exhibit increased cardiac hypertrophy compared to WT mice [16], similar to *Adipo^−/−^* mice. Similarly, the size of infarctions in hearts of mice subjected to ischemia-reperfusion is greater in *T-cad^−/−^* than WT mice [16]. Furthermore, the ameliorative effects of adiponectin in these models are not observed in *T-cad^−/−^* mice [16].

The purpose of this study was to examine the hypothesis that T-cadherin is required for the anti-inflammatory effects of adiponectin that limit the pulmonary inflammation induced by subacute O_3_. To address this hypothesis, we assessed pulmonary inflammation in *T-cad^−/−^* mice and their WT controls exposed to either air or O_3_ (0.3 ppm) for 72 hours. For comparison we also examined *Adipo^−/−^* mice and their WT controls. T-cadherin functions not only as an adiponectin binding protein, but also as a cell-cell adhesion molecule that can impact cell polarization, migration, adhesion, and survival [14]. Hence, effects of T-cadherin deficiency on responses to O_3_ may be the result of the cell-adhesion rather than the adiponectin-binding properties of T-cadherin. To address this issue, we also examined mice deficient in both T-cadherin and adiponectin (*Adipo^−/−^/T-cad^−/−^* mice). We reasoned if effects of T-cadherin deficiency were a reflection of adipnectin binding to T-cadherin, then we would not see any difference between T-cadherin deficient mice and mice deficient in both adiponectin and T-cadherin.

## Methods

### Animals

This study was approved by the Harvard Medical Area Standing Committee on Animals under protocol number 03078 and carried out in accordance with the recommendations in the Guide for the Care and Use of Laboratory Animals from the National Institute of Health. All efforts were made to minimize suffering. *Adipo^−/−^* and *T-cad^−/−^* mice were obtained from Dr. Matsuzaka (Osaka, Japan). *T-cad^−/−^* mice [17] and *Adipo^−/−^* were bred together to obtain *Adipo^−/−^/T-cad^−/−^* mice as previously described [18]. Others have reported small but potentially significant genetic differences in C57BL/6 mice from different vendors [19]. Differences in the microbiome between C57BL/6 mice from Jackson Laboratories and Taconic Farms have also been reported [20]. Hence, as controls for the Adipo^−/−^ mice, we used C57BL/6 from Jackson Laboratories (WT-jax) because this was the genetic background for the Adipo^−/−^ mice. As controls for the *T-cad^−/−^* and *Adipo^−/−^/T-cad^−/−^* mice, we used C57BL/6 mice from Taconic Farms (WT-tac) because this was the background for the *T-cad* ^−/−^ mice. Note that we generated the *Adipo^−/−^/T-cad^−/−^* mice by backcrossing offspring from *Adipo^−/−^*×*T-cad^−/−^* matings onto *T-cad^−/−^* mice [18].

### Protocol

Age and gender matched *Adipo^−/−^*, *T-cad^−/−^*, *Adipo^−/−^T-cad^−/−^*, and control mice were exposed to either air or O_3_ (0.3 ppm, 72 hours) as previously described [8]. Our experience is that neutrophil recruitment plateaus after 48 h of exposure, but significant changes in BAL macrophages do not occur in wildtype mice until 72 h of exposure [8]. Our previous data also indicate that statistically significant changes in BAL macrophages do not occur in wildtype mice until 72 h of exposure [8]. Immediately after the exposure, mice were euthanized by i.p. overdose of sodium pentobarbital. Two cohorts of mice were used. In the first, blood was drawn, the trachea was cannulated to perform bronchoalveolar lavage (BAL), and lungs were stored at −80°C for extraction of total RNA. In the second cohort, lungs were fixed for histological assessment of O_3_-induced pulmonary lesions [21].

### Ozone exposure

Mice were exposed to either O_3_ (0.3 ppm) or ambient air for 72 hours as previously described [8]. Briefly, cages without the microisolator cover were placed in a steel and plexiglass chamber, and supplied with a mixture of O_3_ and air. O_3_ was produced by passing medical grade oxygen through a high voltage ozonizer and bled into the chamber. O_3_ concentration in the chamber was controlled by regulating the amount of ambient air flowing into the chamber. Mice were supplied with normal chow and water *ad libitum*.

### Bronchoalveolar lavage and serum

BAL was performed using two instillations of 1 mL of cold PBS. BAL samples were centrifuged, supernatants were assessed for inflammatory cytokines, and BAL cells were resuspended and counted using a hemocytometer. Cytospin was performed for differential cell analysis. Blood was drawn by cardiac puncture to obtain serum.

### Cytokines and Chemokines

A panel of 32 cytokines, chemokines and growth factors (eotaxin, G-CSF, GM-CSF, IFNγ, IL-1α, IL-1β, IL-2, IL-3, IL-4, IL-5, IL-6, IL-7, IL-9, IL-10, IL-12p40, IL-12p70, IL-13, IL-15, IL-17, IP-10, KC, LIF, LIX, MCP-1, M-CSF, MIG, MIP-1α, MIP-1β, MIP-2, RANTES, TNF-α, and VEGF) were quantified in BAL by a multiplex assay (Eve Technologies, Alberta, Canada) as previously described [22] and a commercial ELISA was used for quantification of sTNFR1 and adiponectin (R&D Systems, MN).

### RNA generation and qRT-PCR

Total lung RNA was extracted using the protocol provided in the RNAeasy kit (Qiagen, MD), and quantified by Nanodrop (ThermoScientific, NJ). cDNA was synthesized from RNA by using a commercial kit [8]. Real time PCR (RT-PCR) was used to assess the mRNA expression of IL-17A, serum amyloid A3 (*saa3*), and *Ki67* (a marker of cell proliferation), by the SYBR green method. Primer sequences are provided in Table 1. Gene expression was normalized to 18S (internal control) followed by analysis by the ΔΔCT method.

**Table 1:** Primers sequence used for qRT-PCR.

Gene	Sense	Anti-sense
18S	5′-GTAACCCGTTGAACCCCATT-3′	5′-CCATCCAATCGGTAGTAGCG-3′
IL-17A	5′-CCAGGGAGAGCTTCATCTGT-3′	5′-AGGAAGTCCTTGGCCTCAGT-3′
ki67	5′-AGGGTAACTCGTGGAACCAA-3′	5′-GGAGGTGAAAACCACACTGG-3′
saa3	5′-CCTGGGCTGCTAAAGTCATC-3′	5′-CACTCATTGGCAAACTGGTC-3′

### Histology

Mice were euthanized and lungs inflated to 20 cmH_2_O with 4% PBS buffered paraformaldehyde (pH = 7.4) overnight. Lungs were washed with PBS and transferred to 70% ethanol, and dehydrated. The left lung was sectioned, embedded in paraffin, and stained with hematoxylin and eosin. The severity of lesions located at terminal bronchioles was assessed by scoring the number of cellular layers below the epithelium as follows: 0 for no lesions, 1: 1–2 cellular layers, 2: for 3 cellular layers, 3: for 4 cellular layers, and 4: for 5 cells or more layers. Total score for each mouse was computed by averaging the scores of all terminal bronchioles present on all sections of left lung.

### Statistical Analysis

The significance of differences between groups was assessed using Factorial ANOVA combined with LSD Fisher as post-hoc analysis (Statistica Software, Statsoft, OK). Data that were not normally distributed were log transformed before statistical analysis. Means and standard errors from log values were retrocalculated with appropriate error propagations. P<0.05 (two tail) was considered significant. Because the controls for the *Adipo^−/−^* and the *T-cad^−/−^* and *Adipo^−/−^/T-cad^−/−^* mice were different, the data from these mice were analyzed separately. All values are expressed as mean±standard mean of error.

## Results

### BAL and serum adiponectin

**Figure 1:**
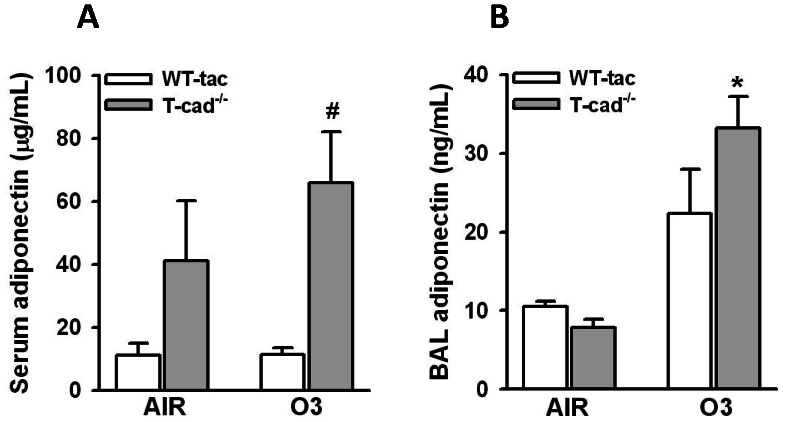
Serum and BAL adiponectin. Total adiponectin was measured by ELISA in serum (A) and bronchoalveolar lavage (BAL) fluid (B) of wildtype (Taconics) and T-cadherin deficient (*T-cad^−/−^*) mice exposed to air and ozone (O_3_, 0.3 ppm) for 72 h. * p<0.05 versus genotype matched air exposed mice; #p0.05 versus wildtype mice with the same exposure. Results of adiponectin in BAL are expressed as mean ± SEM of data from 5 mice exposed to air per group and 7 ozone exposed mice per group. The number of mice used for measurements of serum adiponectin was 4 mice per group.

Serum adiponectin was substantially higher in *T-cad^−/−^* than WT mice (Fig. 1A), consistent with previous observations [15], [17], [18]: adiponectin bound to T-cadherin on the endothelium serves as a repository for adiponectin that is delivered back to the circulation in *T-cad^−/−^* mice [16], [17], [23]. There was no effect of O_3_ exposure on serum adiponectin (Fig. 1A). In contrast, O_3_ exposure caused a marked increase in BAL adiponectin in *T-cad^−/−^* mice (Fig. 1B), likely as a result of increased transit from the blood into the lung consequent to O_3_-induced increases in the permeability of the alveolar/capillary barrier, as previously discussed [8]. BAL adiponectin was slightly lower in air exposed *T-cad^−/−^* versus WT mice and slightly higher in O_3_ exposed *T-cad^−/−^* versus WT mice although these changes were not significant (Fig. 1B).

### Effect of T-cadherin deficiency on O_3_-induced pulmonary inflammation

**Figure 2:**
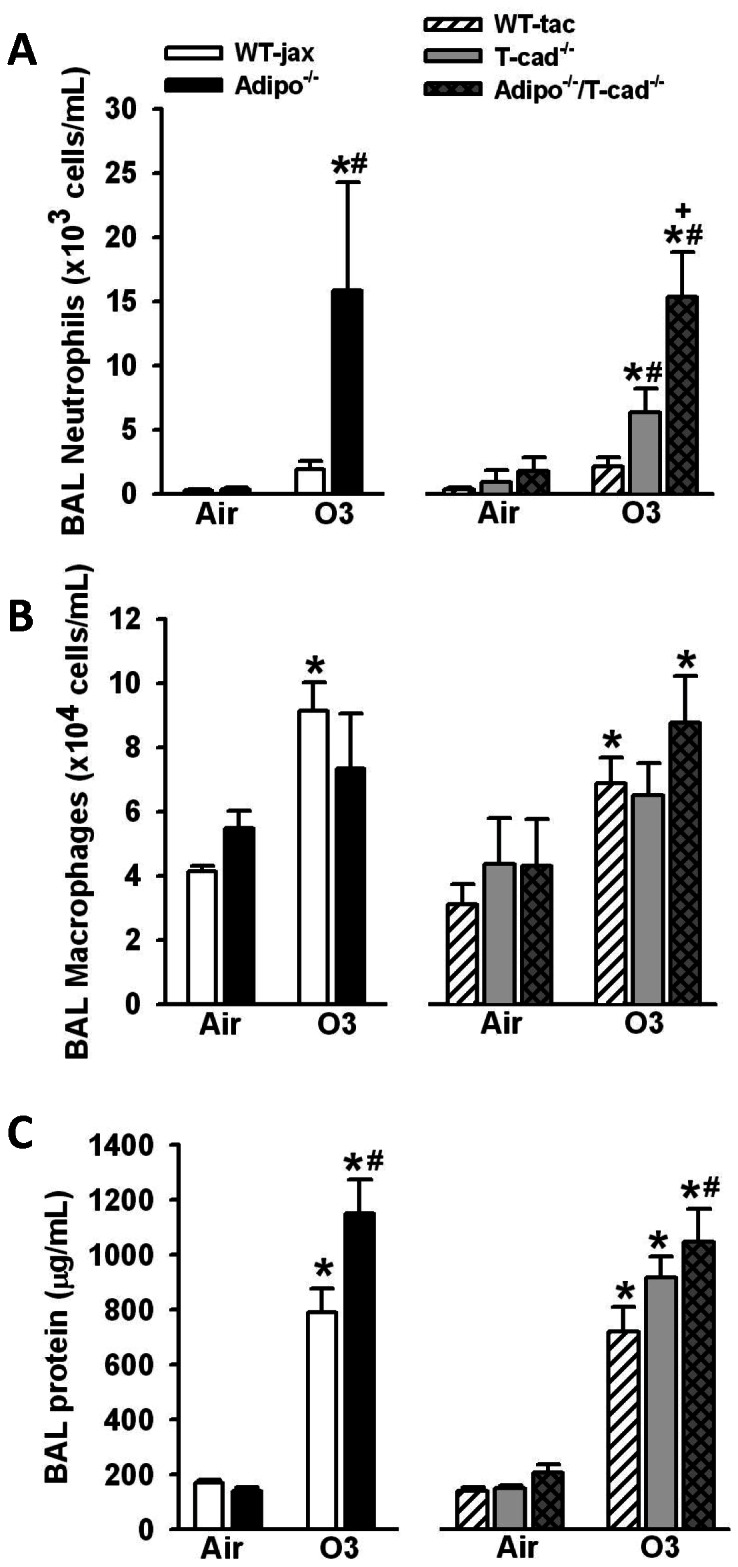
Lung inflammation and injury. BAL neutrophils (A), macrophages (B) and protein (C) in mice exposed to room air or O_3_ (0.3 ppm) for 72 hours. *p<0.05 versus air exposed mice of the same genotype, #p<0.05 versus wildtype mice with the same exposure, + p<0.05 versus T-cadherin deficient mice with the same exposure. Results are mean ± SEM of data from 4–7 air exposed mice and 6–10 ozone exposed mice.

Compared to air, factorial ANOVA demonstrated that O_3_ increased BAL neutrophils, macrophages, and protein (Fig. 2), consistent with previous reports by ourselves and others using this type of O_3_ exposure [8], [24]. BAL neutrophils and protein were higher in O_3_-exposed *Adipo^−/−^* versus WT mice, consistent with our previous observations [8]. Note that BAL neutrophils are also significant greater in *Adipo^−/−^* versus WT mice after 24 or 48 h of exposure [8]. We also observed significantly more BAL neutrophils in O_3_-exposed *T-cad^−/−^* mice versus their corresponding WT controls. BAL neutrophils were also higher in O_3_-exposed *Adipo^−/−^/T-cad^−/−^* than *T-cad^−/−^* mice. No significant change in BAL protein was observed in *T-cad^−/−^* mice exposed to O_3_ compared to WT mice under the same exposure (p = 0.06), despite a trend towards an increase in *T-cad^−/−^* mice. Interestingly, BAL protein was significantly greater in O_3_-exposed *Adipo^−/−^/T-cad^−/−^* versus WT mice (p = 0.005). There was no significant effect of genotype on ozone induced changes in BAL macrophages (Fig. 2B). Note that it is not possible to directly compare *Adipo^−/−^/T-cad^−/−^* and *Adipo^−/−^* mice due to differences in the background strain (see methods).

**Figure 3:**
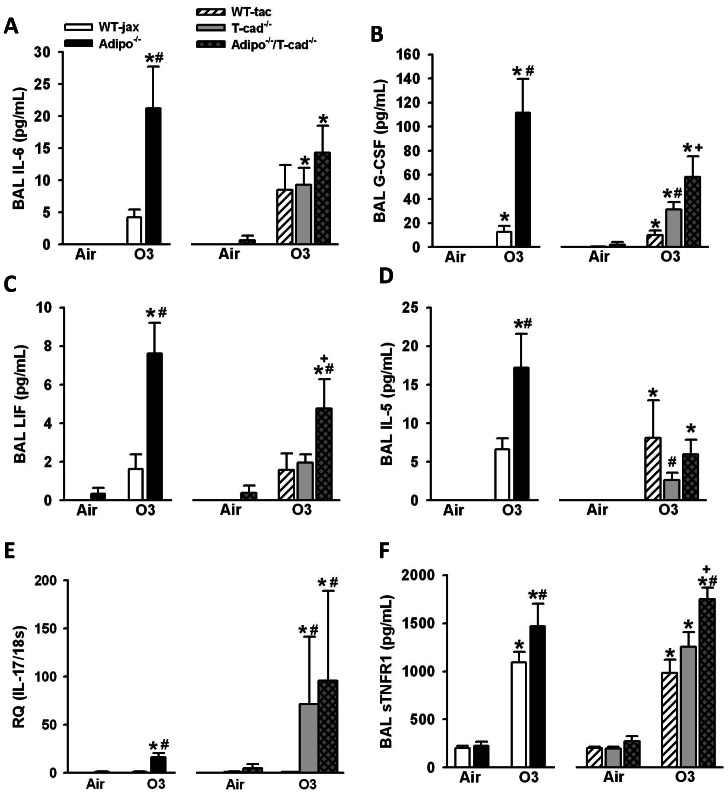
Cytokine and chemokine expression. BAL IL-6 (A), G-CSF (B), LIF (C), IL-5 (D), pulmonary IL-17A mRNA expression (E), and soluble TNFR1 (sTNFR1) (F) in mice exposed to room air or O_3_ (0.3 ppm) for 72 hours. *p<0.05 versus air exposed mice of the same genotype, #p<0.05 versus wildtype mice with the same exposure, + p<0.05 versus T-cadherin deficient mice with the same exposure. Results are mean ± SEM of data from 3–5 air exposed mice and 3–7 ozone exposed mice for IL-6, G-CSF, LIF, and IL-5; 4–9 air exposed and 4–9 for ozone exposed mice for IL-17A mRNA; and 4–7 air exposed and 6–10 ozone exposed mice for sTNFR1;.

To further evaluate the impact of T-cadherin deficiency on O_3_ induced inflammation, we performed a multiplex assay of cytokines and chemokines. Of the factors assayed by multiplex, factorial ANOVA indicated a significant effect of O_3_ exposure in both cohorts of mice (*WT/Adipo^−/−^* and *WT/T-cad^−/−^/Adipo^−/−^/Tcad^−/−^*) for G-CSF, IL-5, IL-6, LIF, KC, and eotaxin. BAL IL-6 and G-CSF were significantly higher in *Adipo^−/−^* versus WT mice exposed to O_3_ (Fig. 3A, B), consistent with our previous observations [8]. We also observed significantly higher BAL LIF and IL-5 in O_3_-exposed *Adipo^−/−^* versus WT mice (Fig. 3C, D). BAL G-CSF was also significantly higher in *T-cad^−/−^* versus WT mice exposed to O_3_, and higher still in *Adipo^−/−^/T-cad^−/−^* versus *T-cad^−/−^* mice (Fig. 3B). Surprisingly, O_3_-induced changes in BAL IL-5 were significantly reduced by T-cadherin deficiency, and this change was reversed by combined adiponectin and T-cadherin deficiency. Neither BAL IL-6 nor LIF was significantly affected by T-cadherin deficiency, although there was significantly greater BAL LIF in O_3_-exposed *Adipo^−/−^/T-cad^−/−^* versus WT mice and a similar trend for IL-6. There was no genotype effect for either eotaxin or KC (data not shown). IL-17A was below the limit of detection of the Bioplex assay, but IL-17A mRNA expression was induced by subacute O_3_ exposure. O_3_-induced increases in IL-17A were significantly greater in *Adipo^−/−^* than WT mice (Fig. 3E), consistent with our previous observations [8]. O_3_-induced increases in IL-17A were also significantly greater in *T-cad^−/−^* versus WT mice, and there was no further increase in *Adipo^−/−^/T-cad^−/−^* versus *T-cad^−/−^* mice.

We also measured BAL concentrations of sTNFR1 (soluble TNFα receptor 1), the extracellular domain of TNFR1 (Fig. 3F). sTNFR1 is cleaved from cell surfaces by the enzyme TACE (TNFα converting enzyme). TACE activity is increased by conditions associated with oxidative stress [25]. Compared to air, O_3_ exposure resulted in a marked increase in BAL sTNFR1 in all mouse genotypes used in this study (Fig. 3F). BAL sTNFR1 was significantly greater in O_3_-exposed *Adipo^−/−^* versus WT mice, consistent with previous observations [8]. There was no significant difference in BAL sTNFR1 in O_3_-exposed *T-cad^−/−^* versus *WT* mice. However, BAL sTNFR1 was significantly higher in O_3_-exposed *Adipo^−/−^/T-cad^−/−^* mice than in either *WT* or *T-cad^−/−^* mice. The results are consistent with the hypothesis that adiponectin deficiency results in increased oxidative stress leading to greater TACE activation, and that T-cadherin is not involved in this effect of adiponectin.

### Histology

**Figure 4:**
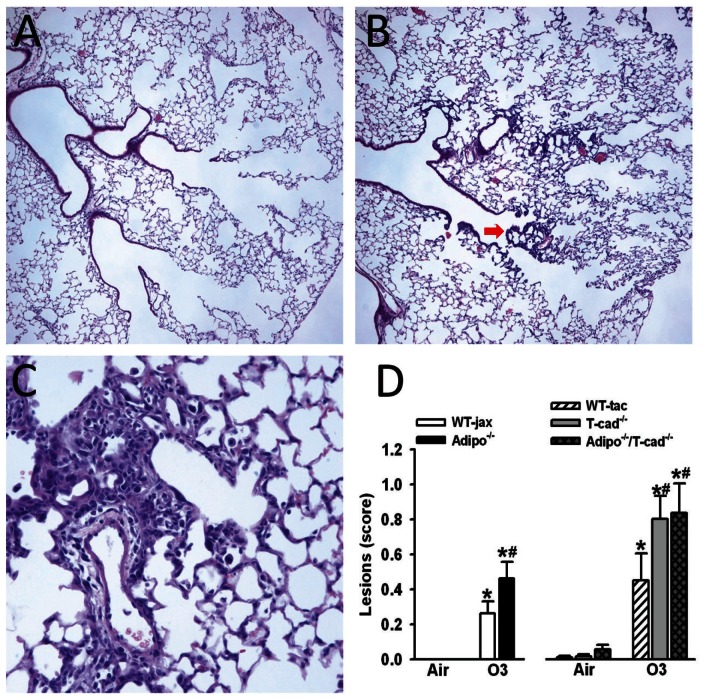
Terminal bronchiolar lesions. H&E stained histological sections of lungs of wildtype mice exposed to air (A) or ozone (B) showing bronchiolar/central acinar lesions in ozone exposed mice (magnification 100×). Arrow in red is pointing a terminal bronchiole with lesion. (C) A detail of these lesions in an ozone exposed mouse (magnification 400×). (D) An average lesion score was calculated for each mouse as described in Methods and these were averaged across mice from each genotype. * p<0.05 versus air exposed mice of the same genotype, # p<0.05 versus wildtype mice with the same exposure. Results are mean ± SEM of data from 6–12 air exposed mice and 8–16 ozone exposed mice.

In O_3_-exposed mice, histopathologic examination revealed inflammatory lesions localized at bronchiolar branch points. These lesions were characterized by focal interstitial expansion by mononuclear cells and reactive hyperplasia of epithelial cells (see Fig. 4B arrow, and a higher magnification of a different airway in Fig. 4C). The severity of the lesions varied, and was quantified as described in Methods. Compared to air, O_3_ exposure resulted in a significant increase in terminal bronchiolar lesions (Fig. 4D). O_3_-induced lesions were significantly greater in *Adipo^−/−^* versus WT mice. O_3_-induced lesions were also significantly greater in *T-cad^−/−^* versus WT mice, but there was no further augmentation in *Adipo^−/−^/T-cad^−/−^* versus *T-cad^−/−^* mice (Fig. 4D).

### qRT-PCR

**Figure 5:**
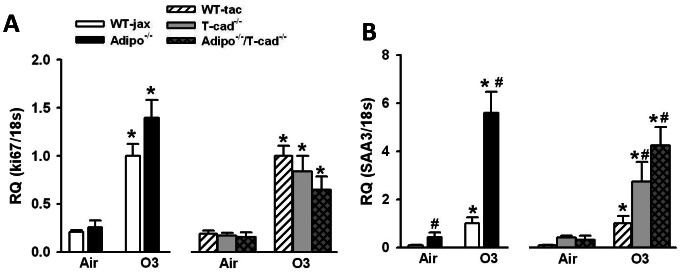
Real time PCR. Pulmonary mRNA expression of ki67 (A) and saa3 (B) in lungs of mice exposed to room air or O_3_ (0.3 ppm ) for 72 h. Expression was normalized to 18 s and expressed relative to wildtype mice exposed to O_3_. *p<0.05 versus air exposed mice of the same genotype, #p<0.05 versus wildtype mice with the same exposure. Results are mean ± SEM of data from 4–7 air exposed mice and 6–10 ozone exposed mice.

To further evaluate the role of T-cadherin in adiponectin dependent effects on O_3_-induced inflammation, we examined the mRNA expression of *Ki67* and *saa3*. We chose to examine *Ki67* because it is a well-established marker of cell proliferation [26]. Others have reported BrdU labeling within the terminal bronchiolar epithelium after O_3_ exposure [27] consistent with epithelial repair following injury, and the lesions we observed in mice exposed to O_3_ included epithelial cell hyperplasia (Fig. 4). We chose to examine *saa3* because others have reported that it is increased by O_3_ to a greater extent in lungs of other types of O_3_-sensitive versus O_3_-resistant mice [28], [29] qRT-PCR data indicated a robust induction of *Ki67* and *saa3* by O_3_ (Fig. 5). O_3_-induced increases in *Ki67* were not affected by either adiponectin or T-cadherin deficiency (Fig. 5A). O_3_-induced expression of *saa3* was higher in *Adipo^−/−^* versus WT mice (Fig. 5B). O_3_-induced expression of *saa3* was also significantly greater in *T-cad^−/−^* than WT mice, and there was no further increase in *Adipo^−/−^/T-cad^−/−^* versus *T-cad^−/−^* mice.

## Discussion

Adiponectin deficiency augments the pulmonary inflammation induced by subacute O_3_ exposure in mice [8], indicating an anti-inflammatory role for adiponectin. Results from the current study show that T-cadherin, an adiponectin binding protein, is required for aspects of this anti-inflammatory effect of adiponectin.

We have previously reported that BAL concentrations of adiponectin increase following subacute O_3_ exposure and that O_3_-induced neutrophilic influx into the lungs is augmented in mice deficient in adiponectin [8]. These results indicate an anti-inflammatory role for adiponectin during subacute ozone exposure. We have also demonstrated that the augmented neutrophilia observed in *Adipo^−/−^* mice is the result of increased IL-17A expression and consequent G-CSF production [8]. The goal of this study was to determine whether T-cadherin, an adiponectin binding protein, contributes to these anti-inflammatory effects of adiponectin. Although several other adiponectin binding proteins have been described [9], [30], we chose to examine T-cadherin because we have shown, using CHO cells overexpressing T-cadherin, that T-cadherin primarily binds the hexameric and high molecular weight (HMW) isoforms of adiponectin [10], the isoforms most abundant in the lung lining fluid [15]. T-cadherin has also been shown to bind adiponectin *in vivo*: T-cadherin is prominently expressed on the apical surface of endothelial cells and immunohistochemistry indicates strong binding of adiponectin to these cells in wildtype mice. In contrast, this binding is lost in *T-cad^−/−^* mice [17]. In addition, T-cadherin is required for the protective effects of adiponectin against cardiac hypertrophy induced by pressure overload and against cardiac injury induced by ischemia-reperfusion [31]. Our results indicate that T-cadherin deficiency mimics aspects of the effects of adiponectin deficiency. After subacute O_3_, *T-cad^−/−^* mice, like *Adipo^−/−^* mice, had increased BAL neutrophils and G-CSF, increased pulmonary IL-17A mRNA expression, as well as increased terminal bronchiolar lesions compared to wildtype mice (Fig. 2A,3B,3E, and 4D), suggesting that binding to T-cadherin is required for effects of adiponectin on these outcomes. In contrast, O_3_-induced increases in BAL protein, a marker of lung injury, O_3_-induced increases in sTNFR1, a marker of oxidative stress, and O_3_-induced increases in BAL IL-6 and LIF were augmented in *Adipo^−/−^* versus wildtype mice, but not in *T-cad^−/−^* versus wildtype mice (Figs. 2,3). IL-6 is required for recruitment of neutrophils after subacute ozone [2], [32]. The role of LIF has not been established, but may be similar to IL-6 since it is a member of the same family of cytokines and shares signal transduction pathways with IL-6 [33]. The results indicate that adiponectin-dependent changes in sTNF1, IL-6, and LIF involve adiponectin acting through other adiponectin binding proteins such as AdipoR1, AdipoR2, and calreticulin [9], [30], or through non receptor mediated effects of adiponectin [34].

**Figure 6:**
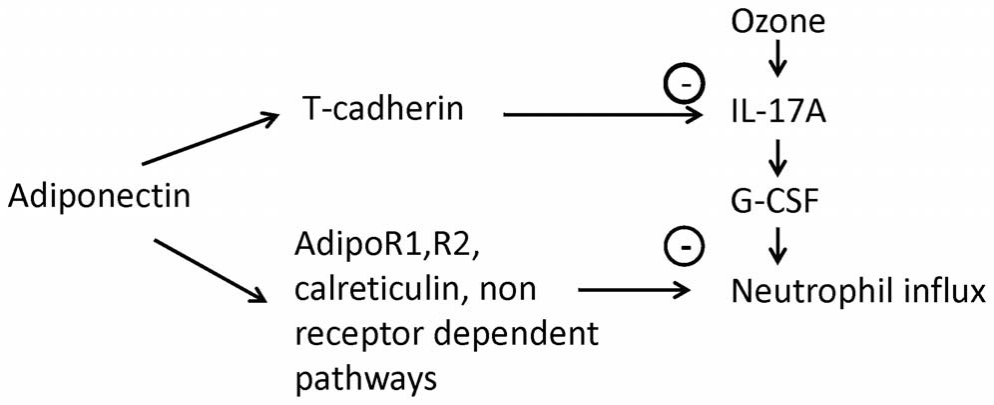
Schematic representation of T-cadherin dependent and independent effects of adiponectin that act to inhibit ozone induced neutrophil influx into the lungs.

In addition to its role as an adiponectin binding protein, T-cadherin has other functions. It acts as a binding protein for lipoproteins [10], [35], [36], [37] and also plays an important role in neuron growth [38] and in polarization and migration of endothelial cells [14]. T-cadherin also reduces surfactant protein D secretion in a pulmonary epithelial cell line [11]. It is possible that loss of such functions, rather than loss of the ability of T-cadherin to bind adiponectin, accounts for the observed effects of T-cadherin deficiency in O_3_ exposed mice. To test this hypothesis, we also examined *Adipo^−/−^/T-cad^−/−^* mice. Although we did not assess adiponectin binding to T-cadherin in this study, our results suggest that adiponectin binding to T-cadherin is required for the ability of adiponectin to inhibit O_3_-induced IL-17A and *saa3* expression, as well as the development of terminal bronchiolar lesions. O_3_-induced IL-17A mRNA expression, terminal bronchiolar lesions, and *saa3* expression, were augmented in *T-cad^−/−^* mice, but no further augmentation was observed in mice with combined adiponectin and T-cadherin deficiency mice (Fig. 3E,4D,5B), indicating that the presence of T-cadherin was necessary for the ability of adiponectin to inhibit these outcomes. In contrast, compared to mice deficient in T-cadherin alone, combined adiponectin and T-cadherin deficiency further augmented BAL neutrophils and G-CSF (Figs. 2A, 3B). Augmented ozone-induced increases in BAL neutrophils and G-CSF are partially dependent on IL-17A [8]. Taken together with the observation that the increased IL-17A expression in *Adipo^−/−^* mice requires T-cadherin (Fig. 3E), the results are consistent with the hypothesis that the augmented O_3_-induced increases in BAL neutrophils and G-CSF in *T-cad^−/−^* versus wildtype mice derive from increases in IL-17A, whereas the additional effects of combined adiponectin and T-cadherin deficiency are the result of adiponectin acting through non-IL-17A dependent pathways (Figure 6).

In contrast to the increased inflammatory responses observed in *T-cad^−/−^* versus wildtype mice after subacute O_3_ exposure reported here, we have previously reported *reduced* airway inflammation in *T-cad^−/−^* versus wildtype mice after allergen sensitization and challenge [18]. In that study, we concluded that T-cadherin does not mediate the effects of adiponectin. Instead, we suggested that the effects of T-cadherin deficiency may result from augmented circulating adiponectin in *T-cad^−/−^* mice (Fig. 1A) acting on other adiponectin binding proteins on circulating or lymphoid tissue Th2 lymphocytes, a key effector cell for allergic airway responses. As discussed above, T-cadherin is highly expressed in endothelial cells [14], where it appears to act as a repository for adiponectin. In the absence of T-cadherin, this adiponectin is delivered back to the blood resulting in increased circulating concentrations [15], [16], [17], [18], [23], as observed (Fig. 1A). One explanation for the divergent effects of T-cadherin deficiency in that study [18] versus this one is as follows. As discussed above, T-cadherin does appear to at least partially mediate the effects of adiponectin that reduce inflammation induced by subacute O_3_. In contrast, other adiponectin binding proteins appear to mediate the anti-inflammatory effects of adiponectin after allergen challenge [18]. The difference in adiponectin binding proteins employed by adiponectin in the two models is likely related to the cell types involved in the two different types of pulmonary inflammation. Whereas CD4^+^ lymphocytes are critical for allergic airways inflammation, the response to subacute O_3_ mainly involves the innate immune response, particularly epithelial cells, macrophages, and pulmonary γδ T-cells [8].

In wildtype mice, we observed an increase in BAL concentrations of the Th2 cytokine, IL-5 after subacute O_3_ exposure (Fig. 3D). Others have also reported an increase in BAL IL-5 after O_3_ exposure in mice, albeit using a different ozone exposure protocol [39]. The role of IL-5 in mediating responses to O_3_ has not been established. In contrast to the effects of T-cadherin deficiency on other O_3_-induced changes in the lung, BAL IL-5 was reduced in T-cadherin deficient mice and restored in adiponectin/T-cadherin bideficient mice. This response is similar to the effects of T-cadherin deficiency on Th2 cytokines in allergen sensitized and challenged mice described above [18], suggesting that the source of IL-5 after O_3_ may be Th2 cells.

Despite the increased circulating concentrations of adiponectin in *T-cad^−/−^* mice (Fig. 1A), we and others [15], [23] have reported reduced BAL adiponectin in naïve *T-cad^−/−^* versus wildtype mice, similar to the results of this study (Fig. 1B, air exposed mice). Such observations indicate that in unchallenged mice, adiponectin is not transported into the lung via simple diffusion: if accumulation of BAL adiponectin relied solely on diffusion, it would be greater in *T-cad^−/−^* versus WT mice, since these mice have greater serum adiponectin (Fig. 1A). Instead, we suggested that T-cadherin may serve to transport adiponectin across the alveolar capillary barrier [15]. Such a hypothesis is consistent with the observation that HMW adiponectin, the adiponectin isoform most readily bound to T-cadherin [10], is also the major isoform present in BAL fluid of naïve mice, whereas the trimeric isoform, which should diffuse most easily, is barely detectable [15], [23]. Loss of such a transport function for T-cadherin is unlikely to explain the effects of *T-cad^−/−^* observed in this study since BAL concentrations of adiponectin were actually greater in O_3_ exposed *T-cad^−/−^* versus wildtype mice (Fig. 1B). Subacute O_3_ exposure results in a marked increase in the permeability of the lungs (Fig. 2C), consistent with lung injury. Based on previous observations indicating that trimeric adiponectin accounted for the majority of the increased BAL adiponectin after ozone [8], we reasoned that in the setting of increased lung permeability, diffusion rather than T-cadherin-mediated transport begins to dominate movement of adiponectin from the blood into the lungs.

Others have demonstrated epithelial injury in mice exposed to O_3_ in this manner, especially in the terminal bronchioles and central acinus, as evidenced by increased BrdU incorporation into these cells, likely reflecting cell proliferation after injury [27], [40], [41]. Consistent with these observations, RT-PCR confirmed increased O_3_-induced expression of one of these genes, *Ki67* (Fig. 5A), a common marker of cell proliferation [26]. However, neither adiponectin deficiency nor T-cadherin deficiency had any effect on *Ki67* mRNA expression (Fig. 5A), suggesting that adiponectin does not regulate cell proliferation in this model. In contrast, we observed effects of both adiponectin deficiency and T-cadherin deficiency on the extent of terminal bronchiolar lesions observed after subacute O_3_ (Fig. 4). Furthermore, adiponectin/T-cadherin bideficiency did not further augment the effects of T-cadherin deficiency on these lesions (Fig. 4), indicating that the effects of T-cadherin deficiency were the result of loss of adiponectin binding to this receptor. Taken together, the results suggest that the impact of adiponectin on terminal bronchiolar lesions is the result of more inflammatory cell recruitment to these sites of injury rather than more epithelial proliferation.

*Saa3* is an acute phase protein capable of recruiting monocytes and macrophages to sites of inflammation, perhaps by forming a complex with the extracellular matrix protein, hyaluronan [42]. O_3_-induced increases in *saa3* mRNA expression were substantially (almost 6 fold) greater in Adipo^−/−^ versus WT mice (Fig. 5B). T-cadherin deficiency also resulted in a marked increase in *saa3* expression in O_3_-exposed mice, and no further augmentation was observed in mice with combined adiponectin and T-cadherin deficiency (Fig. 5B), indicating that the presence of T-cadherin was necessary for adiponectin to suppress *saa3* mRNA expression, similar to our observations with IL-17A (Fig. 3E). We have previously reported that interstitial macrophages and γδ T cells are the source of the augmented IL-17A produced in the lungs following subacute O_3_ exposure in *Adipo^−/−^* mice [8]. *Saa3* has the capacity to induce IL-17A expression in T cells [43] and it is possible that it also regulates IL-17A expression in lung after subacute O_3_ exposure.

One technical issue requires discussion. We exposed mice to O_3_ for 72 hours. Others have shown gene specific differences in the kinetics of gene expression following O_3_ [28], [29]. Hence, it is likely that cytokines and chemokines other than those we identified in Fig. 3 as being impacted by O_3_ were induced at earlier times in the exposure and then declined. Moreover, it is possible that the earlier expression of these moieties contributes to responses observed after 72 h exposure. However, we have examined the time course of key outcomes described here that differ in WT versus Adipo^−/−^ mice (BAL neutrophils, IL-17 mRNA expression) and have found that these genotype-related differences exist throughout the 72 h exposure period described here[8].

In summary, our results confirm an anti-inflammatory role for adiponectin in pulmonary responses to subacute O_3_ and indicate that adiponectin binding to T-cadherin is required for aspects of this response, including the induction of IL-17A and consequent recruitment of neutrophils to the lungs.
